# Sobering Overview of Traumatic Craniofacial Injuries Involving Drugs and Alcohol: A Comprehensive Analysis of the NEISS Database

**DOI:** 10.3390/cmtr18010013

**Published:** 2025-02-07

**Authors:** Sam Boroumand, Ismail Ajjawi, Tara Boroumand, Omar Allam, Lioba Huelsboemer, Viola A. Stögner, Martin Kauke-Navarro, Siba Haykal, Michael Alperovich, Bohdan Pomahac

**Affiliations:** 1 Department of Surgery, Division of Plastic & Reconstructive Surgery, Yale University School of Medicine, New Haven, CT 06510, USA; ismail.ajjawi@yale.edu (I.A.); omar.allam@yale.edu (O.A.); lioba@huelsboemer.de (L.H.); viola.stogner@yale.edu (V.A.S.); kauke-navarro.martin@yale.edu (M.K.-N.); siba.haykal@yale.edu (S.H.); michael.alperovich@yale.edu (M.A.); bohdan.pomahac@yale.edu (B.P.); 2 San Francisco School of Dentistry, University of California, San Francisco, CA 94115, USA; tara.boroumand@ucsf.edu; 3 Department of Plastic, Aesthetic, Hand and Reconstructive Surgery, Hannover Medical School, Burn Center, 30625 Hannover, Germany

**Keywords:** alcohol, craniofacial, drugs, injury, maxillofacial, trauma

## Abstract

The purpose of this study is to provide a comprehensive overview of the epidemiology of traumatic craniofacial injuries in the U.S. that occur in the context of drugs and/or alcohol. The National Electronic Injury and Surveillance System (NEISS) database was queried from 2019 to 2022 to identify all emergency department (ED) patients with reported traumatic injuries involving craniofacial anatomy. Clinical and demographic variables were assessed between cases that involved drugs/alcohol and those that did not, utilizing two-proportion z-tests. In total, 441,764 patients with traumatic craniofacial injuries were identified, with 20,285 cases involving drugs and/or alcohol. Cases involving drugs/alcohol had a significantly higher frequency of internal injuries (45.3% vs. 34.9%, *p* < 0.001), fractures (7.0% vs. 3.4%, *p* < 0.001), and hematomas (4.6% vs. 2.5%, *p* < 0.001) than those that did not. Craniofacial injuries involving drugs/alcohol also had a significantly higher frequency of falls to the floor (19.2% vs. 11.8%, *p* < 0.001) and down stairs/steps (18.3% vs. 6.6%, *p* < 0.001) and resulted in a significantly greater odds of hospital admission after ED presentation (OR 3.75, 95% CI: 3.63–3.88, *p* < 0.001). Ultimately, these epidemiological findings can be leveraged to provide clinicians invaluable qualitative context for the most frequent presentations of craniofacial injuries they can anticipate to treat among intoxicated patients.

## 1. Introduction

Alcohol and drug use results in a total cost of over USD 420 billion annually, with USD 120 billion in healthcare costs [1]. Aside from the financial burden that results from these substances, their misuse compromises public health and safety. A 2015 study found that in the previous year, 61 million people in the United States reported binge drinking, and 44 million abused a non-prescribed or illicit drug [1,2]. Alcohol and other drugs are a commonly recognized risk factor in the occurrence of traumatic injuries, with the prevalence of these substances in Level 1 trauma centers estimated to be as high as 71% [3]. Binge alcohol drinking is a common risk factor for craniofacial injuries due to its contributions to road traffic injuries, falls, and assaults [4]. A study that evaluated 129 craniofacial injury patients in the ICU found alcohol consumption to be a statistically significant denominator in these cases [5]. Other studies that have analyzed the etiology of facial trauma have likewise identified the significant role of alcohol and substance use in these injuries due to its contribution to interpersonal violence and motor accidents [6,7,8].

To date, studies evaluating the impact of drugs and alcohol with craniofacial injuries have been limited in scope or patient population evaluated [9,10]. By utilizing a national, multicenter database, our study aims to provide a comprehensive overview of the epidemiology encompassing drug and alcohol use with traumatic craniofacial injuries. Through this detailed analysis, we seek to enhance the understanding of how alcohol and other drugs contribute to the frequency and severity of craniofacial injuries and hope to not only inform targeted preventative measures for promoting safer substance use practices but also provide clinicians invaluable qualitative context for the most frequent presentations of craniofacial injuries they can anticipate to treat among intoxicated patients.

## 2. Methods

### 2.1. Dataset and Study Cohort

We performed a cross-sectional study utilizing The National Electronic Injury and Surveillance System (NEISS) database to identify patients with craniofacial injuries who were seen in the emergency department (ED) from 1 January 2019 to 31 December 2022. Institutional IRB approval was not required for this publicly available, de-identified patient dataset. The Consumer Product Safety Commission maintains the NEISS database to keep a record of all non-fatal traumatic injuries seen in the ED from 100 hospitals that form a nationally representative sample. The NEISS database contains information on patient demographics, diagnosis, location of injury, ED disposition, and a brief explanation about the context of the injury. To study the impact of drugs and/or alcohol on craniofacial injury patterns presenting to the ED, individuals with classified craniofacial injuries involving injuries to the head (NEISS BDYPT code 75), face (NEISS BDYPT code 76), eyeball (NEISS BDYPT code 77), mouth (NEISS BDYPT code 88), neck (NEISS BDYPT code 89), and ear (NEISS BDYPT code 94) were extracted from the database. This identified patient population was then divided into two comparison groups based on whether there was any alcohol and/or drug involvement noted with the injury. Alcohol involvement was recorded if the patients admitted to alcohol use or were intoxicated at the time of their ED evaluation. Drug involvement was recorded if the patients admitted to the use of or were found to be under the influence of illicit mood-altering substances at the time of their ED evaluation. The specifics of which illicit or non-prescribed substances that were utilized in each patient case were not provided.

### 2.2. Study Variables and Data Analysis

The variables evaluated in the analysis included sex, age, race/ethnicity, type of craniofacial injury, injury location, consumer products involved, and ED dispositional outcome. All statistical analyses were performed using R Studio (version 3.0.3). Descriptive statistics were reported as a frequency and percentage for categorical variables and median and interquartile range for continuous variables. To compare proportions of various craniofacial injury variables, two-proportion z-tests were used. To compare the likelihood of hospital admission between injuries with drug/alcohol involvement and injuries without drug/alcohol involvement, the odds ratios were calculated with 95% confidence intervals reported. *p*-values of less than 0.05 were considered statistically significant. 

## 3. Results

### 3.1. Study Cohort/Demographics

**Figure 1:**
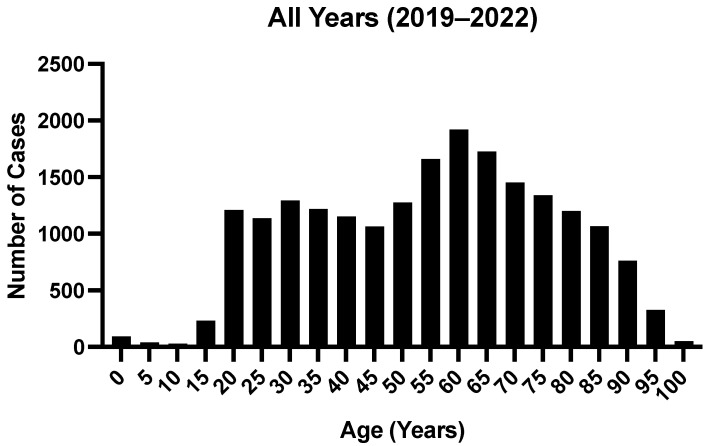
Age demographic distribution of craniofacial injuries involving drugs/alcohol identified in NEISS 2019–2022.

We identified 441,764 patients in total who sustained craniofacial injuries from 2019 to 2022 in the NEISS database. From the 441,764 patients identified, 20,285 of the identified cases involved drugs and/or alcohol, while 421,479 patients did not. The number of cases involving drugs/alcohol was extrapolated to a weighted national approximation of 860,321 patients when using the sampling weights assigned by the NEISS database, which are dependent on the geographic location of the injury. The age, sex, and race/ethnicity characteristics of the study population are highlighted in Table 1. For cases involving drugs/alcohol, the majority of craniofacial injuries occurred in males (61.3%) and individuals of a white racial background (53.6%). Figure 1 provides a distribution of the ages of patients presenting with craniofacial injuries that had drug/alcohol involvement from 2019 to 2022.

### 3.2. Trends of Type of Injury and Consumer Products Involved

As shown in Table 2, the most common type of injury in both craniofacial cases that had drug/alcohol involvement and cases that did not involve drug/alcohol involvement was internal injury (intracranial injuries to the brain, such as edema, contusions, or bleeding), with drug/alcohol cases incurring a significantly higher frequency between the two groups (45.3% vs. 34.9%, *p* < 0.001). Craniofacial injuries without drug/alcohol involvement were more likely to be lacerations (24.9% vs. 22.6%, *p* < 0.001) and foreign bodies (4.2% vs. 0.1%, *p* < 0.001), while injuries with drug/alcohol involvement were more likely to be fractures (7.0% vs. 3.4%, *p* < 0.001) and hematomas (4.6% vs. 2.5%, *p* < 0.001). The most common cause of injury in both patient populations was falls to floors, with cases involving drugs/alcohol incurring a higher frequency than injuries with no drug/alcohol involvement (19.2% vs. 11.8%, *p* < 0.001). Among the other most common causes of craniofacial injury, cases with drug/alcohol involvement had a significantly higher frequency of falls down stairs/steps (18.3% vs. 6.6%, *p* < 0.001), bicycles and accessories (4.9% vs. 2.9%, *p* < 0.001), ceilings and walls (3.9% vs. 2.8%, *p* < 0.001), while craniofacial injuries without drug/alcohol involvement had significantly higher rates of involvement of beds/bedframes (8.8% vs. 6.5%, *p* < 0.001), tables (3.7% vs. 3.3%, *p* < 0.001), couches (2.4% vs. 0.9%, *p* < 0.001), and jewelry (2.2% vs. 0.06%, *p* < 0.001).

### 3.3. Trends of Injury Outcomes and Incident Locations

As highlighted in Table 3, patients presenting to the ED with craniofacial injuries involving drugs/alcohol were more frequently hospitalized relative to patients presenting with cases that had no drug/alcohol involvement (25.3% vs. 8.3%, *p* < 0.001). On the other hand, patients presenting with injuries that had no drug/alcohol involvement were more frequently treated and discharged from the ED (87.8% vs. 66.2%, *p* < 0.001). According to Table 4, the odds ratio of admission for cases involving drugs/alcohol compared to cases with no drug/alcohol involvement was 3.75 (95% CI is 3.63–3.88). This ratio was slightly higher for males (4.02, 95% CI is 3.85–4.19) and slightly lower for females (3.42, 95% CI is 3.24–3.61). In the ED, cases involving drugs/alcohol had a significantly higher frequency of being held for observation (3.1% vs. 0.7%, *p* < 0.001), leaving without being seen (2.8% vs. 2.1%, *p* < 0.001), being treated and transferred to a different hospital (2.4% vs. 0.9%, *p* < 0.001), and experiencing mortality (0.1% vs. 0.06%, *p* = 0.009). The most common location for both types of injuries was at home, with higher frequency for the drug/alcohol cases (49.2% vs. 45.6%, *p* < 0.001). However, drug/alcohol cases were more likely to occur in public (16.4% vs. 8.8%, *p* < 0.001) and on the street (5.6% vs. 2%, *p* < 0.001). On the other hand, craniofacial injuries with no drug/alcohol involvement were significantly more likely to occur in school (4.7% vs. 0.3%, *p* < 0.001) and during sports (9.6% vs. 1.9%, *p* < 0.001).

### 3.4. Sex Differences in Craniofacial Injuries Involving Drugs/Alcohol

Table S1 shows the sex differences within the patient population presenting with craniofacial injuries that involved drugs/alcohol. Males in this population experienced a significantly higher frequency of lacerations (24.5% vs. 19.6%, *p* < 0.001) and fractures (7.7% vs. 5.8%, *p* < 0.001), while females were significantly more likely to experience internal injuries (47.4% vs. 43.9%, *p* < 0.001) and hematomas (6.6% vs. 3.3%, *p* < 0.001). Regarding causes of injury, females were more likely to be injured with falls to the floor (22.3% vs. 17.3%, *p* < 0.001), beds (8.2% vs. 5.4%, *p* < 0.001), tables (3.7% vs. 3.1%, *p* = 0.01), and bathtubs (3.7% vs. 2.5%, *p* < 0.001). Following ED admission, males were more likely to be treated and admitted/hospitalized (25.9% vs. 24.4%, *p* = 0.01) and to leave without being seen (3.1% vs. 2.3% *p* < 0.001), while females were more likely to be treated/examined and released (67.5% vs. 65.3%, *p* = 0.001). Regarding the location of injury, females were more likely to be injured at home (56.7% vs. 44.5%, *p* < 0.001) while males were more likely to be injured during sports (2.4% vs. 1.3%, *p* < 0.001) and on the street (7.7% vs. 2.4%, *p* < 0.001).

### 3.5. Hospitalization Differences in Craniofacial Injuries Involving Drugs/Alcohol

Table S2 shows the differences between craniofacial injuries that included drug/alcohol involvement and those that did not within the patient population that was hospitalized following ED admission. Craniofacial injuries that involved drugs/alcohol and resulted in hospital admission were more likely to be internal injuries (59% vs. 56.6%, *p* = 0.001), and lacerations (11% vs. 8.8%, *p* < 0.001), while craniofacial injuries without drug/alcohol involvement that resulted in hospital admission were more likely to be fractures (12.7% vs. 10.9%, *p* < 0.001) and burns (1.4% vs. 1%, *p* = 0.04). Regarding causes of injury, craniofacial injuries that involved drugs/alcohol and resulted in hospital admission were more likely to be caused by falls down stairs or steps (20.6% vs. 9.5%, *p* < 0.001) and ceilings or walls (2.8% vs. 2.2%, *p* = 0.01), while injuries without drug/alcohol involvement that resulted in hospital admission were more likely to be caused by falls to the floor (27.4% vs. 23.6%, *p* < 0.001), beds (10.8% vs. 8%, *p* < 0.001), and toilets (3.3% vs. 2.4%, *p* < 0.001).

## 4. Discussion

In the face of America’s escalating substance and alcohol use crisis, the impact on craniofacial injuries emerges as a stark testament to its far-reaching consequences [11,12]. This comprehensive analysis, one of the largest studies to date involving over 400,000 patients, provides insights into the severity and epidemiological factors of craniofacial injuries associated with alcohol and substance use. These findings offer a critical clinical context for healthcare professionals and public health officials, laying the groundwork for the development of targeted preventative measures to address this pressing issue.

Our study reveals that patients under the influence of drugs or alcohol with craniofacial injuries were nearly four times more likely to be hospitalized compared to those without substance involvement. This finding is consistent with the published literature, as multiple smaller studies have shown similar results [9,10]. The effect of alcohol and drug use on hospitalization rates is multiplex and likely correlated to higher acuity and more severe craniofacial trauma. Internal craniofacial injuries were significantly more common in individuals under the influence of drugs or alcohol, with almost half of the patients suffering internal injuries. Although the specific internal injuries in each case were not explicitly provided by the database, which can range from brain bleeds to cerebral contusions, this finding serves as a proxy for a higher severity of traumatic injuries.

The impact of drugs and alcohol on the severity of craniofacial trauma is multifaceted. Factors such as increased pain tolerance, increased aggression, decreased inhibition, and impaired cerebral function all contribute to higher-energy and more severe traumas [13,14,15,16,17,18,19,20,21,22]. When a patient falls with impaired coordination, the result is often more significant craniofacial damage. This is because the facial structure is one of the furthest points from the center of gravity, resulting in the largest potential energy and moment of inertia during a fall [23]. Without appropriate hand–eye coordination to brace for the impact, the face absorbs a substantial portion of the traumatic force [24].

Gender differences were also noted, with men showing a higher frequency of hospitalization following substance-related craniofacial injuries. This disparity likely stems from men’s tendency to engage in riskier behaviors and consume larger quantities of substances when under the influence, as well as their greater BMI resulting in larger traumatic force [25,26,27]. Moreover, the physiological effects of drugs and alcohol, such as depressed respiratory drive, aspiration risk, and impaired neurological function, further complicate emergency care and require more frequent hospitalizations and advanced medical interventions [28,29,30].

A key finding of our study is that the most common causes of substance-related craniofacial injuries were falls down stairs and at ground level, often occurring in public spaces and on streets. This discovery presents a significant public health opportunity. While previous research has demonstrated an increased risk of substance use and falls, our data confirm this as the primary cause of craniofacial trauma in the United States [31,32,33,34,35]. The prevalence of these injuries in public spaces underscores the potential for effective public health policies. While individual-level interventions have shown substantial benefits in reducing various types of injuries, our findings suggest that broader community-based initiatives and regulatory policies could play a critical role in mitigating injury risks associated with problem drinking [36]. Potential strategies include enforcing public health standards to prevent overserving in social settings, implementing community education programs, limiting alcohol sales in public spaces, creating safer environments for alcohol and drug use, and modifying public spaces to reduce fall risks, such as safer steps and sidewalks in front of bars.

The healthcare system faces challenges in addressing substance use disorders among trauma patients. Studies show that a significant percentage of patients with alcohol- and drug-related issues fail to seek treatment due to insufficient recognition and inadequate referral by trauma care providers [37,38,39,40,41,42]. Moreover, there has been a concerning decline in drug testing among trauma patients, despite an increase in positive results over recent years [41]. Brief alcohol interventions and counseling have proven effective in reducing alcohol consumption and associated trauma recidivism [43,44]. Surgeons treating traumatic craniofacial injuries in this patient population are uniquely positioned to use these interactions as teachable moments, discussing the dangers of substance use and working to prevent future incidents [45]. By utilizing the findings of this epidemiological study to guide their patient education, craniofacial trauma surgeons can provide direct, impactful insights to reduce the risk of repeat trauma injuries among their patients that frequently use drugs and alcohol.

## 5. Limitations

A few limitations of this study should be noted. More granular clinical details regarding each ED admission for craniofacial injury that could have provided additional context to the findings outlined in this study were not available from the NEISS database, including the exact extent/severity of the craniofacial injury, the type or amount of drugs and/or alcohol intoxication of the patient, as well as any treatment interventions or operative care performed. Additionally, the NEISS database is sourced from data collected over many healthcare systems and hospitals across the country. As a result, there may be bias or potential errors in the reporting of data from these individual hospitals. We must also be cognizant that because the NEISS database only captures injuries from ED admissions, this study cannot characterize the true incidence/prevalence of craniofacial injuries resulting from drug/alcohol use if the patients did not present to the ED or alternatively presented to an outpatient clinic following their injury. With these considerations in mind, it is important to note that the U.S. Consumer Product Safety Commission and the Center for Disease Control have supported the NEISS database’s capacity to provide nationally representative data of traumatic injuries treated in EDs within the U.S. [46].

## 6. Conclusions

Based on a national, multicenter sample, this study effectively provides an epidemiological overview of craniofacial injuries compounded by drug/alcohol use. The likelihood of hospitalization following craniofacial injury is nearly four-fold greater under the influence of drugs and alcohol, with a significantly greater frequency of specific injury types/etiologies. Ultimately, these epidemiological findings can be leveraged to not only inform targeted preventative measures for promoting safer substance use practices but also provide clinicians invaluable qualitative context for the most frequent presentations of craniofacial injuries they can anticipate to treat among intoxicated patients.

## Figures and Tables

**Table 1:** Demographic overview of craniofacial injuries identified in NEISS database with and without drug/alcohol involvement.

	Injuries with Drugs/Alcohol (*n* = 20,285)	Injuries Without Drugs/Alcohol (*n* = 421,479)
Average age (SD)	57 (37–71)	14 (4–56)
Sex		
Male	12,439 (61.3%)	228,618 (54.2%)
Female	7843 (38.6%)	192,845 (45.8%)
Race/Ethnicity		
White	10,876 (53.6%)	193,199 (45.8%)
Black/African American	2833 (13.9%)	63,824 (15.1%)
Asian	223 (1%)	8480 (2%)
American Indian/Alaska Native	117 (0.5%)	1294 (0.3%)
Native Hawaiian/Pacific Islander	23 (0.1%)	582 (0.1%)
Other	304 (1.5%)	14,493 (3.4%)
Unknown	5909 (29.1%)	139,607 (33.1%)

**Table 2:** Comparison of types and causes of injuries between craniofacial injuries with and without alcohol/drug involvement.

	Injuries with Drugs/Alcohol (*n* = 20,285)	Injuries Without Drugs/Alcohol (*n* = 421,479)	*p*-Value
Type of Injury			
Internal Injury	9183 (45.3%)	147,122 (34.9%)	<0.001
Laceration	4594 (22.6%)	105,244 (24.9%)	<0.001
Contusions	2083 (10.2%)	43,593 (10.3%)	0.734
Fracture	1424 (7.0%)	14,710 (3.4%)	<0.001
Hematoma	932 (4.6%)	10,741 (2.5%)	<0.001
Concussion	549 (2.7%)	24,848 (5.9%)	<0.001
Foreign Body	32 (0.1%)	17,969 (4.2%)	<0.001
Top Causes of Injury			
Floors	3906 (19.2%)	50,093 (11.8%)	<0.001
Stairs or Steps	3716 (18.3%)	27,939 (6.6%)	<0.001
Beds or Bedframes	1320 (6.5%)	37,154 (8.8%)	<0.001
Bicycles and Accessories	997 (4.9%)	12,502 (2.9%)	<0.001
Ceilings and Walls	803 (3.9%)	11,949 (2.8%)	<0.001
Chairs	681 (3.3%)	14,232 (3.4%)	0.880
Tables	671 (3.3%)	15,753 (3.7%)	<0.001
Bathtubs or Showers	601 (2.9%)	11,238 (2.6%)	0.010
Couches	192 (0.9%)	10,157 (2.4%)	<0.001
Jewelry	13 (0.06%)	9368 (2.2%)	<0.001

**Table 3:** Comparison of ED disposition and locations of injury between craniofacial injuries with and without alcohol/drug involvement.

	Injuries with Drugs/Alcohol (*n* = 20,285)	Injuries Without Drugs/Alcohol (*n* = 421,479)	*p*-Value
ED Disposition			
Treated/examined and released	13,416 (66.1%)	370,026 (87.8%)	<0.001
Treated and admitted/hospitalized	5143 (25.3%)	34,942 (8.3%)	<0.001
Held for observation	631 (3.1%)	3286 (0.7%)	<0.001
Left without being seen	578 (2.8%)	8894 (2.1%)	<0.001
Treated and transferred	494 (2.4%)	4052 (0.9%)	<0.001
Fatality	23 (0.1%)	274 (0.06%)	0.009
Unknown	0 (0%)	5 (0.001%)	0.623
Incident Location			
Home	9984 (49.2%)	192,499 (45.6%)	<0.001
Public	3334 (16.4%)	37,209 (8.8%)	<0.001
Street	1144 (5.6%)	8627 (2.0%)	<0.001
Sports	405 (1.9%)	40,349 (9.6%)	<0.001
School	70 (0.3%)	20,035 (4.7%)	<0.001
Mobile	8 (0.03%)	62 (0.01%)	0.006
Farm	6 (0.02%)	166 (0.04%)	0.489
Industry	3 (0.01%)	37 (0.01%)	0.379
Unknown	5331 (26.3%)	122,495 (29.1%)	<0.001

**Table 4:** Odds ratios for hospital admission following craniofacial injury involving drug/alcohol use.

	Odds Ratio	95% Confidence Interval	*p*-Value
Drugs/Alcohol Craniofacial Injury—Total	3.75	3.63–3.88	<0.001
Drugs/Alcohol Craniofacial Injury—Males	4.02	3.85–4.19	<0.001
Drugs/Alcohol Craniofacial Injury—Females	3.42	3.24–3.61	<0.001

## Data Availability

The publicly available NEISS dataset can be procured at the following link: https://www.cpsc.gov/Research--Statistics/NEISS-Injury-Data (accessed on 10 March 2024).

## Supplementary Materials

The following supporting information can be downloaded at https://www.mdpi.com/article/10.3390/cmtr18010013/s1: Table S1. Sex differences among craniofacial injuries with drug/alcohol involvement; Table S2. Comparison of most common types and causes of craniofacial injuries.
