# Supplementary material for: Sobering Overview of Traumatic Craniofacial Injuries Involving Drugs and Alcohol: A Comprehensive Analysis of the NEISS Database
Source: Craniomaxillofac Trauma Reconstr. 2025 Feb 7;18(1):13. doi: 10.3390/cmtr18010013 (PMC11995830; doi:10.3390/cmtr18010013)
Supplement: Supplementary file 1 [file cmtr-18-00013-s001.zip › cmtr-3370557-supplementary.pdf]

Supplementary Table 1. Sex differences among craniofacial injuries with drug/alcohol involvement

	Male (n= 12,439)	*Female (n=7,843)*	*p-value*
Type of Injury			
Internal Injury	5,465 (43.9 %)	3,717 (47.4 %)	**<0.001**
Laceration	3,054 (24.5 %)	1,540 (19.6 %)	**<0.001**
Contusions	1,307 (10.5 %)	776 (9.9 %)	0.161
Hematoma	413 (3.3 %)	519 (6.6 %)	**<0.001**
Fracture	967 (7.7 %)	457 (5.8 %)	**<0.001**
Concussion	298 (2.4 %)	251 (3.2 %)	**<0.001**
Top Causes of Injury			
Stairs or Steps	2,312 (18.5 %)	1,403 (17.8 %)	0.210
Floors	2,152 (17.2 %)	1,754 (22.3 %)	**<0.001**
Bicycles and Accessories	865 (6.9 %)	132 (1.7 %)	**<0.001**
Beds	677 (5.4 %)	643 (8.2 %)	**<0.001**
Ceilings and Walls	505 (4.0 %)	298 (3.8 %)	0.354
Chairs	414 (3.3 %)	267 (3.4 %)	0.769
Tables	380 (3.1 %)	291 (3.7 %)	**0.011**
Bathtubs	309 (2.5 %)	292 (3.7 %)	**<0.001**
ED Disposition			
Treated / Examined and Released	8,121 (65.3 %)	5,293 (67.5 %)	**0.001**
Treated and Admitted / Hospitalized	3,227 (25.9 %)	1,916 (24.4 %)	**0.015**
Treated and Transferred	309 (2.5 %)	185 (2.3 %)	0.572
Held for observation	374 (3.0 %)	257 (3.3 %)	0.280
Left Without Being Seen	394 (3.1 %)	183 (2.3 %)	**<0.001**
Died	14 (0.1 %)	9 (0.1 %)	0.963
Unknown	0 (0 %)	0 (0 %)	**NA**
Incident Location			
Home	5,537 (44.5 %)	4,447 (56.7 %)	**<0.001**
School	35 (0.28 %)	34 (0.4 %)	0.069
Public	2,094 (16.8 %)	1,240 (15.8 %)	0.055
Sports	303 (2.4 %)	102 (1.3 %)	**<0.001**

Street	954 (7.7 %)	190 (2.4 %)	**<0.001**
Farm	4 (0.03 %)	2 (0.02 %)	0.788
INDST.	2 (0.01 %)	1 (0.01 %)	0.849
Mobile	7 (0.05 %)	1 (0.01 %)	0.128
Unknown	3,503 (28.1 %)	1,826 (23.3 %)	**<0.001**

Supplementary Table 2. Comparison of most common types and causes of craniofacial injuries with and without alcohol/drug involvement among patients who were admitted to hospital.

	Injuries with Drugs/Alcohol (n=5,143)	*Injuries without Drugs/Alcohol (n=34,942)*	*p-value*
Types of Injury			
Internal Injury	3,036 (59.0 %)	19,786 (56.6 %)	**0.001**
Laceration	567 (11.0 %)	3,078 (8.8 %)	**<0.001**
Fracture	565 (10.9 %)	4,455 (12.7 %)	**<0.001**
Contusions	353 (6.8 %)	2,202 (6.3 %)	0.123
Hematoma	206 (4.0 %)	1,126 (3.2 %)	0.003
Concussion	56 (1.1 %)	934 (2.7 %)	**<0.001**
Burns	53 (1.0 %)	477 (1.4 %)	**0.049**
Top Causes of Injury			
Floors	1,214 (23.6 %)	9,580 (27.4 %)	**<0.001**
Stairs or Steps	1,062 (20.6 %)	3,315 (9.5 %)	**<0.001**
Beds	415 (8.0 %)	3,782 (10.8 %)	**<0.001**
Bicycles and Accessories	210 (4.1 %)	1,359 (3.9 %)	0.503
Chairs	159 (3.1 %)	1,265 (3.6 %)	0.055
Bathtubs or Showers	154 (2.9 %)	1,211 (3.4 %)	0.081
Ceilings or walls	143 (2.8 %)	787 (2.2 %)	**0.018**
Toilets	125 (2.4 %)	1,171 (3.3 %)	**<0.001**
